# Preparing Superposition States to Modify the Spectra and to Achieve Complete Selectivity in Photodissociation Reactions

**DOI:** 10.1021/acs.jctc.5c00655

**Published:** 2025-07-30

**Authors:** Ignacio R. Sola, Alberto García-Vela

**Affiliations:** † Departamento de Química Física, 16734 Universidad Complutense de Madrid (y Unidad Asociada I+D+i CSIC), Madrid 28040, Spain; ‡ 16379 Instituto de Física Fundamental, Consejo Superior de Investigaciones Científicas, Serrano, 123, Madrid 28006, Spain

## Abstract

We derive and apply the geometric optimization methodology to modify the photodissociation spectra of CH_3_I in the A band. For this purpose, we prepare optimized initial wave functions that maximally exploit interference-induced coherent control to drive a reaction mediated by nonadiabatic couplings in a polyatomic molecule essentially from the beginning. By designing functionals that maximize the output of the products, or that imply competition between the products, or discrimination of one of them, we test the performance of the methods and the effect of preparing initial vibrational coherences among CH_3_–I stretching vibrational states, CH_3_ vibrational states, or both. Our results show that using weak ultrashort pulses, one can easily increase the efficiency of the reaction toward any of the products by 100–200% using vibrational states related to the reaction coordinate; that one can increase the efficiency by more than 100% and at the same time almost completely quench the output of products in the other channels. Finally, if one demands high selectivity in the reaction, we show that it is possible to suppress even the most dominant channel to less than one part in a million by preparing superpositions of all available vibrational states optimized with the proper functional.

## Graphical abstract

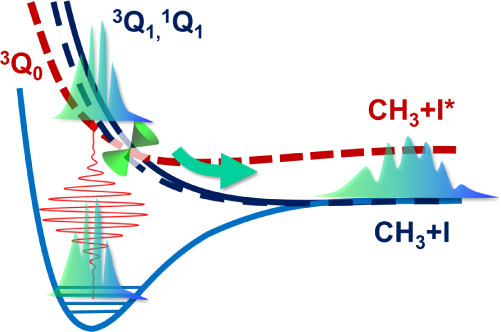

Controlling molecular reaction dynamics has been a subject of increasing interest in recent decades.^1–7^ Much effort has been put into the strong field regime, developing a theoretical and numerical framework^8–14^ to parametrize and to find optimal control strategies^15–19^ and to implement optical schemes with modulated pulses in the laboratory.^20–24^ In the weak-field regime, the spectra are the main observables that encode the effect of the dynamics, so controlling different types of spectra becomes the most important control objective. Indeed, the first formulations of coherent control^25,26^ and optimal control theory^27^ were developed within the formalism of perturbation theory. Substantial understanding has been gained on the role of different masks, pulse modulations, and combinations of delayed pulses.^23,28–32^ It is for instance well-known that no control can be achieved in the asymptotic time limit upon excitation out of a single vibrational state in first-order perturbation theory, that is, in single-photon spectroscopy.^33^ But this is not the case when the initial wave function is a superposition of vibrational states, as first proposed by Brumer and Shapiro.^25^ The preparation of the initial state implicitly requires the use of an additional pulse, which leads to second-order perturbation effects at the minimum.

The geometrical optimization (GO) is a variational methodology that, in its simplest application,^34^ allows to find the optimal initial superposition that maximizes the desired process. It can be seen as an alternative or complementary^35^ control scheme to the usual optimal control theory (OCT), where a functional related to the desired outcome of the dynamics is constructed and its variations are computed with respect to changes in the driving field.^1–7^ While in OCT the complexity lies in the optimal pulse and the optical or electronic coherences are maximally used to control the dynamics, in GO the complexity lies in the initial state, whereas the field is typically an unmodulated ultrashort optical laser. The mechanism behind the GO is based on the interference between quantum pathways starting from different states (codified by the superposition coefficients) that end in the same final state, showcasing the utmost importance of the initial coherences in controlling quantum processes. Ultimately, these ground electronic coherences must be prepared by suitable infrared or optical pulses (via stimulated Raman). The takeaway lesson in GO is that the IR (or Raman) pulses should be applied before the pump pulse drives the population to the excited states, where the dynamics occurs, reverting the typical recipe of starting with the pump pulse followed by the control pulse. It should be noted that, in the context of photochemical reactions, the role of the initial coherences has been related to imparting a kick to the initial wave packet along the required direction to select the desired reaction channel.^36–40^

Previously, the GO has been applied to maximize the yield and rate of absorption,^40–42^ to control isomerization reactions^43^ and to accelerate selective adiabatic passage.^44^ Recently, it has been applied to control the branching ratio of the photodissociation reaction of CH_3_I in the first absorption band^45^ (the A band, ranging from 220 to 350 nm with a maximum at about 260 nm), where the presence of a conical intersection connecting the two main excited valence states produces interesting nonadiabatic effects in the photolysis dynamics. Photodissociation of CH_3_I in the A band has been investigated both experimentally and theoretically, ever since the spectrum was measured almost 50 years ago.^46^ In fact, many pioneering experimental techniques were first tested on discerning the photodissociation dynamics in the A band. This process was the first one investigated by the ion imaging experimental technique by Chandler and Houston^47^ at 266 nm, later followed by velocity map imaging (VMI) measurements by Eppink and Parker^48,49^ over nearly the whole band (240–334 nm). In addition, time-resolved studies by Zewail and coworkers^50^ led to a series of time-resolved VMI experiments for different regions of the A band.^51–54^ More recently, photoelectron imaging experiments in the range 245.5–261.6 nm were also reported.^55^

Regarding the theoretical works, the photolysis dynamics of CH_3_I in the A band was first studied by a two-dimensional wave packet model by Shapiro and Bersohn,^56^ later improved by adding a third degree of freedom related to the H_3_–C–I bending mode^57^ (ν_6_), and wave packet studies including four,^54^ five,^58^ and the full nine^59^ dimensions of the system. Those models used either the six-dimensional,^60^ or the full-dimensional^61^ *ab initio* excited potential energy surfaces of CH_3_I calculated by Morokuma and coworkers, and further refined by Xie et al.^62^

Despite the great deal of works reported in the last decades on the CH_3_I photodissociation, only a few of them have been devoted to the control of this process. Actually, to the best of our knowledge only three theoretical,^25,45,63^ and two experimental^64,65^ quantum control works on the CH_3_I photolysis in the A band have been reported. The two main excited states involved in the CH_3_I photodissociation, namely ^3^ *Q*_0_ and ^1^ *Q*_1_, are connected by a conical intersection and give rise to two different product channels forming CH_3_ + I*­(^2^ *P*_1/2_) and CH_3_ + I­(^2^ *P*_3/2_), respectively (hereafter referred to as the I* and I channels, respectively).

In a previous study,^45^ the quantum yield in the photodissociation of CH_3_I in the I and I* channels was controlled, analyzing the role of the pulse duration and the effect of adding different vibrational states of the CH_3_–I stretching mode (the reaction coordinate) in the optimized initial superposition, working always in the weak-field regime. The aim of the present work is to extend the application of the GO control scheme to different transformations of the spectra, related to increasing the output of its products, maximizing the difference spectra (the contrast between the signals coming from different product channels) and discriminating products achieving high channel selectivity. They will imply deriving the GO equations for different functionals, including the control of the quantum yield and branching ratio. In addition, the effect of other vibrational modes in the optimized superposition will be analyzed, including the vibrational states associated with both the CH_3_–I stretching mode and the CH_3_ group modes in the initial superposition. The main goal of this work is essentially methodological. The analysis of the above points should be very useful to design an experimental application of the control scheme, since it will help to make such designs both simple and efficient.

## Methods and Models

### Model and Spectra

Photodissociation of CH_3_I in the A band involves a dominant parallel ^3^ *Q*_0_ ← *X̃* ^1^ *A*_1_ transition and two rather weak perpendicular transitions, ^1^ *Q*_1_ ← *X̃* ^1^ *A*_1_ and ^3^ *Q*_1_ ← *X̃* ^1^ *A*_1_, of lower intensity. The photodissociation process is schematically depicted in Figure 1. The ^1^ *Q*_1_ and ^3^ *Q*_1_ states correlate asymptotically to the same fragments, CH_3_ + I­(^2^ *P*_3/2_), while ^3^ *Q*_0_ correlates to the CH_3_ + I*­(^2^ *P*_1/2_) products. The asymptote of ^3^ *Q*_0_ is separated from that of ^1^ *Q*_1_ and ^3^ *Q*_1_ by the iodine spin–orbit splitting, 0.943 eV.^48^ In addition, a conical intersection (CI) connects the ^3^ *Q*_0_ and ^1^ *Q*_1_ states, so wave packet amplitude initially excited to any of these two states will bifurcate at the CI, producing both CH_3_ + I and CH_3_ + I* fragments.

**Figure 1:**
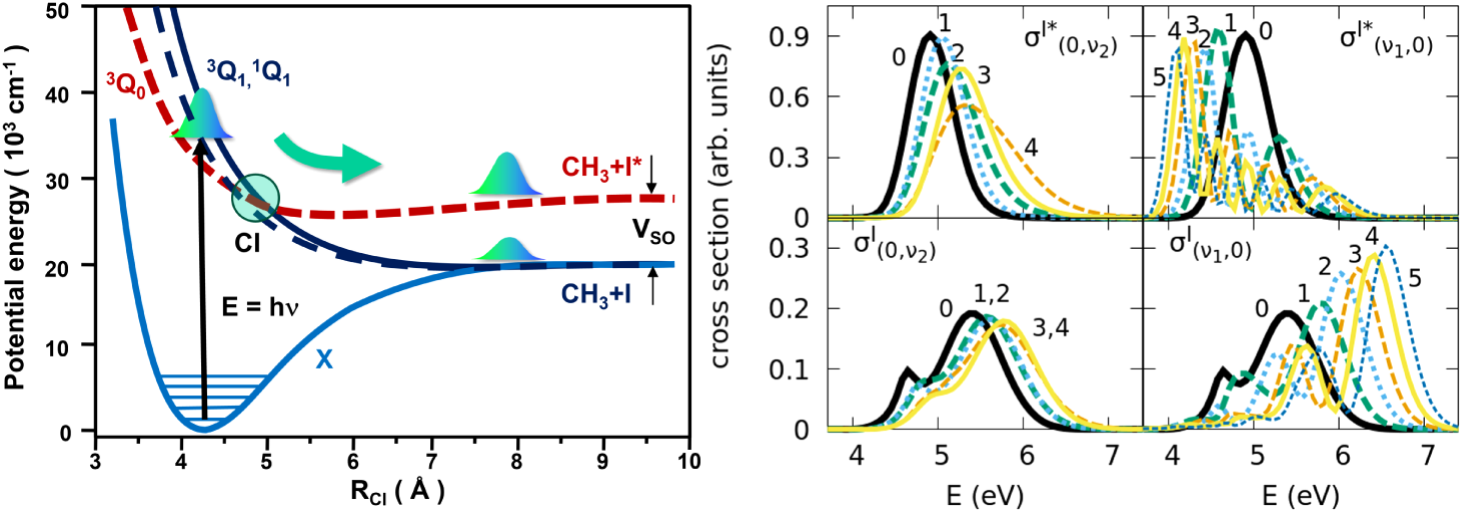
Potential-energy curves along the CH_3_–I coordinate are shown in the left panel for the four electronic states, *X̃* ^1^ *A*_1_, ^3^ *Q*_0_, ^1^ *Q*_1_, and ^3^ *Q*_1_, involved in the photodissociation of CH_3_I in the A band, pictorially indicating the position of the conical intersection (CI). The asymptotes correlated to the two chemical channels, I and I*, are separated by the spin–orbit splitting, *V*_*SO*_. On the right panels we show the spectra as a function of the total energy (kinetic plus internal energy) for the I* fragment (upper panels) and the I fragment (lower panels), for different starting excitations of the CH_3_–I stretching, $\sigma_{\nu_{1},0}^{e}$ , and the CH_3_ vibrational modes, $\sigma_{0,\nu_{2}}^{e}$ (with *e* = *I*, I*). As ν_1_ increases, the peaks of the photospectra are red-shifted for the I* channel, while they are blue-shifted for the I fragment. The first one dominates at low energies, while the second dominates at high energies, particularly at energies quite larger than the nonadiabatic crossing, as high kinetic energy favors the transition. On the other hand, the A bands only widen and shift slightly to the blue as ν_2_ increases, but mostly overlap.

In the dynamical simulations we use a fully quantum three-dimensional model Hamiltonian, in which the methyl iodide is represented as a CXI pseudotriatomic molecule,^57^ where the pseudoatom X = H_3_ is located at the center-of-mass of the three atoms. Jacobi coordinates (*R*,*r*,θ) are used to represent the three degrees of freedom considered in the model, namely the dissociation CH_3_–I coordinate stretching mode (*R*), the C–X distance that represents the umbrella bend of the C–H_3_ group (*r*), and the X–C–I bending mode (θ), which is the angle between the vectors associated with *R* and *r*. The dynamical simulations solve the time-dependent Schrödinger equation, assuming zero total angular momentum (*J* = 0). The wave packet propagation is carried out by using the Chebychev polynomial expansion method using a complex absorbing potential placed at the end of the grid employed.^66^ Details on the model, its validity, the ground^67,68^ and excited^62^ potential energy surfaces (PES), and the transition dipole moment functions^67,68^ used, as well as the wave packet propagation scheme, are described elsewhere^52,53^ and are provided in the Supporting Information. The initial states propagated consisted of the direct product $\varphi_{\nu_{1}}\left(R\right)\rho_{\nu_{2}}\left(r,\theta \right)$ , where $\varphi_{\nu_{1}}\left(R\right)$ and $\rho_{\nu_{2}}\left(r,\theta \right)$ are the vibrational eigenfunctions associated with the CH_3_–I stretching mode and with the CH_3_ group within CH_3_I, with ν_1_ and ν_2_ being the quantum numbers labeling their vibrational states, respectively. The calculation of the $\varphi_{\nu_{1}}\left(R\right)\rho_{\nu_{2}}\left(r,\theta \right)$ vibrational eigenfunctions is described elsewhere.^52^ In the standard notation for CH_3_I, ν_1_ and ν_2_ label the symmetric stretching and the umbrella mode of the CH_3_ group, while ν_3_ labels the CH_3_–I stretching mode. However, for simplicity, we will use the indices ν_1_ and ν_2_ to label the vibrational states of the CH_3_–I stretching mode and of the CH_3_ group, respectively.

The partial photodissociation cross sections are computed by projecting out the asymptotic wave packet onto the corresponding fragment states, as^69,70^

1 $$\sigma_{v}^{e,\nu ,j}\left(E\right)=\frac{C\omega k_{v}^{e,\nu ,j}}{2\pi }{|\int_{0}^{t_{f}}\langle \chi_{\nu }^{\left(j\right)}\left(r\right)P_{j}\left(\cos\theta \right)|\Psi_{e,v}\left(R_{c},r,\theta ,t^{\prime }\right)\rangle e^{iEt^{\prime }}dt^{\prime }|}^{2}$$

where *C* is a constant factor, ω is the incident photon frequency, *e* = 1–3 denotes the three excited electronic states, *v* = (ν_1_,ν_2_) are the initial vibrational states, *R*_*c*_ is a suitably large distance of the dissociation coordinate *R*, *E* is the total energy of the system reached with each excitation wavelength, *E* = *E*_*e*_ + *ℏω* (being *E*_*e*_ the energy of the CH_3_I (ν_1_,ν_2_) initial state), and $k_{v}^{e,\nu ,j}$ is given by

2 $$k_{v}^{e,\nu ,j}={[2m\left(E-V_{\mathrm{SO}}\delta_{e1}-E_{\nu ,j}\right)]}^{1/2}$$

with *V*_SO_ being the spin–orbit splitting between the two lowest electronic states of iodine, and δ_*e*1_ being the Kronecker delta. The eigenstates of the CH_3_ fragment (the C–X pseudodiatomic molecule) are represented by the product $\chi_{\nu }^{\left(j\right)}\left(r\right)P_{j}\left(\cos\theta \right)$ (ν_2_ is a collective index comprising ν and *j*), where $\chi_{\nu }^{(j)}\left(r\right)$ are the rovibrational states of C–X in the umbrella mode (with associated rovibrational energies *E*_ν,*j*_) and *P*_*j*_(cos θ) are Legendre polynomials. Ψ_*e*,*v*_(*R*_*c*_,*r*,θ,*t*′) is the wave packet in the excited electronic state *e* at time *t*′. To calculate the photodissociation cross sections, the relevant energy range *E* containing the A band (3.4–7.7 eV) is represented by a grid of 67 energy points, and the initial superposition is optimized for each of these energies of the grid.

In a previous work,^45^ only the six lowest vibrational states of ν_1_ were included in the initial superposition, while we kept ν_2_ = 0. In the present simulations we add ν_2_ = 0–4 to the set of ν_1_ = 0–5 states from which the spectrum is calculated, so 30 different (ν_1_,ν_2_) initial vibrational states have been propagated. The difference in energy between the lowest and the highest of these 30 vibrational states is ∼4500 cm^–1^. In the current simulations each (ν_1_,ν_2_) vibrational state undergoes a Franck–Condon excitation from *X̃* ^1^ *A*_1_ to the three excited states, which is equivalent to be pumped by an infinitely short Delta pulse.^71^ This is another difference with the earlier simulations,^45^ where Gaussian laser pulses with a finite temporal width were applied. Our results showed that, as long as a sufficient number of vibrational states could participate in the initial wave function, higher yields could be obtained using pulses as short as possible. In practice, as shown in ref 45, it suffices to use a 5 fs pulse (fwhm) of GW/cm^2^ intensity to achieve very high yields in the photodissociation of CH_3_I in the A band, providing a good approximation to the implicit delta pulses that ignite the dynamics of this work.

The wave packet propagation of each (ν_1_,ν_2_) state on the excited electronic states was carried out for a total time *t*_*f*_ = 200 fs, with a time step of 0.4 fs. This propagation time ensures that all the wave packet amplitude reaches the asymptotic region.

The choice of the initial vibrational ν_1_ state has a clear impact in the photodissociation spectra, as the CH_3_–I stretching mode is involved in the bond that dissociates. Since the Franck–Condon transition mainly excites the wave packet in the repulsive ^3^Q_0_ state, which correlates with fragmentation in I*, the photodissociation cross section for this fragment, $\sigma_{v}^{I^{*}}\left(E\right)$ , exhibits the well-known reflection principle, by which the nodal pattern of the initial vibrational state determines the shape of the spectrum^71^ (see the upper right panels of Figure 1). This is particularly noticeable at low energies, below the nonadiabatic crossing. In addition, the peak of the spectra shifts to lower energies for larger ν_1_.

On the other hand, $\sigma_{v}^{I}\left(E\right)$ is the sum of the contribution of the two electronic states, ^3^Q_1_ and ^1^Q_1_, that correlate with fragmentation into I and depends on the nonadiabatic transition from ^3^Q_0_, shown in Figure 1, leading to a binodal distribution in the spectrum from the ground vibrational level, $\sigma_{(0,0)}^{I}\left(E\right)$ . In this case, the peak of the spectra shifts to higher energies for larger ν_2_ (see the lower right panels of Figure 1). The photodissociation spectra in the I channel also reflect the shape of the vibrational wave functions, but only at energies above the nonadiabatic crossing, which are associated with the regions of the wave function closer to the repulsive barrier. This is why the I* fragment dominates at lower energies, while the I fragment dominates at higher energies, especially when the dissociation is fast enough to favor the nonadiabatic transition that brings population from the ^3^Q_0_ state to the ^3^Q_1_ and ^1^Q_1_ asymptotes. But the largest values of $\sigma_{v}^{I}\left(E\right)$ are always quite smaller than the largest values of $\sigma_{v}^{I^{*}}\left(E\right)$ (notice the different scales of the cross sections in Figure 1), since the Franck–Condon factors tend to be smaller at high energies.

Because the nodes of the CH_3_ vibrational states are orthogonal to the reaction coordinate, the shape of these eigenstates is not reflected in the spectra. Excitation of the ν_2_ vibrational modes has a subtler effect: The shape of the photodissociation band in both channels (but more importantly in the I channel) barely changes as ν_2_ increases.

### Control Schemes

In the following, the basic features of the GO procedure are described on which the control scheme applied in this work is based. For notational convenience, we rewrite eq 1 as

3 $$\sigma_{v}^{e,n}\left(E\right)=\frac{C\omega k_{v}^{e,n}}{2\pi }{|\int_{0}^{\infty }\langle \psi_{n}^{e}(E)|Û(t,0)|\varphi_{v}^{g}\rangle e^{iEt}dt|}^{2}$$

where $\psi_{n}^{e}$ is the scattering wave function for electronic channel *e* and total energy *E*, with all vibrational and rotational quantum numbers of the fragments given collectively by *n*, $\varphi_{v}^{g}$ is the initial wave function, similarly with all vibrational levels labeled by the collective index *v*, and *Û*(*t*,0) is the time evolution operator. Developing the square, we can write the expression for the total cross section as the expectation value of an operator

4 $$\sigma_{v}^{e}\left(E\right)\propto \underset{n}{\sum }k_{v}^{e,n}\left\langle \Phi_{v}\left(E\right)\right|{Ŝ}_{e,n}\left|\Phi_{v}\left(E\right)\right\rangle$$

where ${Ŝ}_{e,n}=\left|\psi_{n}^{e}\left(E\right)\right\rangle \left\langle \psi_{n}^{e}\left(E\right)\right|$ is the projection operator over the desired final state of the fragments, and

5 $$\left|\Phi_{v}\left(E\right)\right\rangle =\int_{0}^{\infty }Û\left(t,0\right)\left|\varphi_{v}^{g}\right\rangle e^{iEt}dt$$

is the function containing all the dynamical information on the photodissociation process. Notice that since we are only interested in the cross section of different chemical channels, in eq 4 we sum over all the internal states of fragments, as well as over all the electronic states that dissociate to the same channel, I or I*. So $\sigma_{v}^{I}$ implies summing the expectation values obtained with ${Ŝ}_{2,n}$ + ${Ŝ}_{3,n}$ , where *e* = 2,3 refers to the electronic states ^1^Q_1_ and ^3^Q_1_, respectively. Defining the complex magnitude $A_{v}^{e,n}\left(E\right)=\sqrt{k_{v}^{e,n}}\left\langle \psi_{n}^{e}\left(E\right)\right|\Phi_{v}\left(E\right)\rangle$ , eq 4 becomes $\sigma_{v}^{e}\left(E\right)\propto \underset{n}{\sum }A_{v}^{e,n*}\left(E\right)A_{v}^{e,n}\left(E\right)$ . Now, if instead of a single eigenstate, the initial state is a coherent superposition of *N* vibrational eigenstates of the ground state potential, where different coefficients can be chosen depending on the energy of the fragments (or energy of the spectrum) $\varphi \left(0\right)=\overset{N}{\underset{j}{\sum }}c_{j}\left(E\right)\varphi_{j}^{g}$ , the photodissociation cross section will be proportional to

6 $$\begin{array}{l} \sigma^{e}\left(E\right)\propto \underset{n}{\sum }\underset{j}{\sum }\underset{k}{\sum }c_{j}^{*}\left(E\right)c_{k}\left(E\right)\left\langle \Phi_{j}\left(E\right)\right|{Ŝ}_{e,n}\left|\Phi_{k}\left(E\right)\right\rangle \\ =\underset{n}{\sum }\underset{j}{\sum }\underset{k}{\sum }c_{j}^{*}\left(E\right)c_{k}\left(E\right)A_{j}^{e,n*}\left(E\right)A_{k}^{e,n}\left(E\right)=c^{T}\left(E\right)S^{e}\left(E\right)c\left(E\right) \end{array}$$

where we defined the scattering matrix **S** ^e^(*E*), with elements $S_{jk}^{e,n}\left(E\right)=\left\langle \Phi_{j}\left(E\right)\right|{Ŝ}_{e,n}\left|\Phi_{k}\left(E\right)\right\rangle =A_{j}^{e,n*}\left(E\right)A_{k}^{e,n}\left(E\right)$ , and the superposition coefficients were arranged as a column vector **c**(*E*) (or its conjugate transpose row vector **c** ^T^(*E*)). As long as **S** ^*e*^(*E*) has nondiagonal elements, σ^e^(*E*) will exhibit interference patterns, such that one can maximize the quantum yield over the desired channel by optimizing the initial superposition state.

Equation 6 is a quadratic form. Finding its extrema with respect to variations in vector **c** ^T^(*E*) [or **c**(*E*)] subject to normalization conditions, **c**(*E*)^T^ **c**(*E*) = 1, is a Rayleigh-Ritz problem. We call *F* ^*e*^(**c**) = **c** ^T^ **S** ^*e*^ **c** the functional whose extrema are the eigenvalues of the matrix **S** ^*e*^(*E*). The eigenvector **c**_opt_(*E*) with largest eigenvalue $\sigma_{opt}^{e}\left(E\right)$ gives the coefficients of the initial superposition that maximizes the cross section for channel I or I* at energy *E*. Since $\sigma_{opt}^{e}\left(E\right)$ is proportional to the products obtained in channels I or I* at each energy, these superpositions can be used to maximize particularly desired products at specifically chosen energies.

It is also possible to define other functionals for different goals. We will be concerned with functionals that involve *competition* between the product channels. In the context of Quantum optimal control theory, this problem is often referred to as multitask control^19^ where it is often difficult to find robust solutions. As we show here, in the frame of the geometrical optimization this multitask simply leads to a different eigenvalue equation. For instance, if we want to maximize the total amount of products regardless of the chemical channel, $\sigma^{t}=\sigma^{I}\left(E\right)+\sigma^{I^{*}}\left(E\right)$ , we need to find the extrema of the functional $F^{t}\left(c\right)=c^{T}\left[S^{I}+S^{I^{*}}\right]c$ subject to normalization of **c**, for which we calculate the eigenvalues and eigenvectors of matrix $S^{t}\left(E\right)=S^{I}\left(E\right)+S^{I^{*}}\left(E\right)$ . Because in general the matrices **S** ^*I*^(*E*) and $S^{I^{*}}\left(E\right)$ do not commute, their eigenvectors will be different: superpositions that maximize the sum will typically not maximize neither of the products. Alternatively, we can maximize the difference spectrum, defined as $\sigma^{d}=\sigma^{I}\left(E\right)-\sigma^{I^{*}}\left(E\right)$ , for which we need to obtain the eigenvalues and eigenvectors of the matrix $S^{d}\left(E\right)=S^{I}\left(E\right)-S^{I^{*}}\left(E\right)$ . Clearly, the largest eigenvalue, σ^*d*^_opt_(*E*), will depend on a balance between maximization of σ^*I*^(*E*) and minimization of $\sigma^{I^{*}}\left(E\right)$ .

However, if we really demand to achieve highly selective photodissociation into a specific fragment, it is more useful, as we will show in this work, to maximize the desired quantum yield, which implies maximizing a ratio. To do so, we define a functional of the quantum yield on channel I, *F* ^χ^ = **c** ^T^ **S** ^*I*^ **c**/**c** ^T^ **S** ^*t*^ **c**. Calculating its gradient and imposing constraints over the norm leads to the nonlinear eigenvalue equation^45^

7 $$\frac{1}{\sigma^{t}\left(E\right)}\left[S^{I}\left(E\right)-\chi^{I}\left(E\right)S^{I}\left(E\right)-\chi^{I}\left(E\right)S^{I^{*}}\left(E\right)\right]c\left(E\right)=\lambda c\left(E\right)$$

We obtain the maximum yield from the eigenvector with largest eigenvalue **c**_opt_(*E*),

8 $$\chi_{\mathrm{opt}}^{I}\left(E\right)=\frac{c_{\mathrm{opt}}^{T}\left(E\right)S^{I}\left(E\right)c_{\mathrm{opt}}\left(E\right)}{c_{\mathrm{opt}}^{T}\left(E\right)\left(S^{I}\left(E\right)+S^{I^{*}}\left(E\right)\right)c_{\mathrm{opt}}\left(E\right)}$$

where χ^*I*^ = σ^*I*^/σ^*t*^, and which provides the optimal initial superposition. We call *discriminating* functionals to these quadratic forms that imply ratios. Equivalently, one could design a functional to maximize the branching ratio $\gamma \left(E\right)=\sigma^{I}\left(E\right)/\sigma^{I^{*}}\left(E\right)$ , from which one could derive the nonlinear eigenvalue equation

9 $$\left[S^{I}\left(E\right)-\gamma \left(E\right)S^{I^{*}}\left(E\right)\right]c\left(E\right)=\lambda c\left(E\right)$$

that allows to obtain the optimal branching ratio choosing the proper superposition state (the eigenvector with largest eigenvalue). We have found that eq 9 gives the same values that eq 7, but is numerically more unstable, for which in this work we always optimize the quantum yield. The same procedures could be followed to maximize the quantum yield in channel I*.

## Results and Discussion

### Maximizing the Cross Sections

Our first quest in the control of the photochemical reaction will be to maximize its output products, changing the spectra via geometrical optimization. Then we will use functionals that imply competition between the product channels, and finally we will refer to the selectivity of the process, defining functionals that discriminate between products. In Figure 2 we show results where we optimize the initial state to maximize products I* (upper panels) or I (lower panels), measured as an increase in the photodissociation cross section associated with the desired channel. As a reference for the control performance, we will always show the photodissociation starting from the (ν_1_,ν_2_) = (0,0) ground vibrational state. At typical temperatures in experiments with CH_3_I, this state dominates in the corresponding Boltzmann distribution of vibrational states, so the A band is essentially dominated by the $\sigma_{(0,0)}^{I}$ and $\sigma_{(0,0)}^{I^{*}}$ cross sections, which can be taken as the reference results in the absence of control. Another reference for the performance of the control scheme are the cross sections obtained from the single vibrational state within the three sets of states ((0,ν_2_), (ν_1_,0), and (ν_1_,ν_2_)) that leads to the maximal cross section, which we call σ_max_ ( $\sigma_{max}^{I}$ and $\sigma_{\max}^{I^{*}}$ , correspondingly) or maximal spectra. Comparison of these σ_max_ spectra with the maximal cross sections that can be achieved starting from the variationally optimized superposition state, denoted by σ_opt_ ( $\sigma_{opt}^{I}$ and $\sigma_{\mathrm{opt}}^{I^{*}}$ , for the maximization of channels I and I*, respectively), gives a measure of the importance of interference in the control mechanism. σ_max_ can be thought of as the best possible result that can be obtained as part of a “passive” control mechanism.

**Figure 2:**
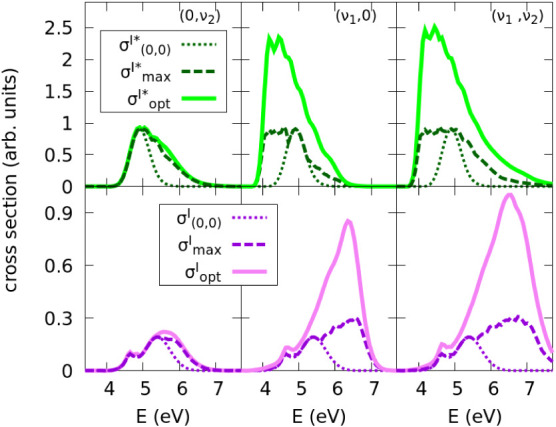
Cross sections of the I* (upper panels) and I (lower panels) fragment channel. The functionals *F* ^*I*^ and $F^{I^{*}}$ are maximized using excited vibrational modes of the CH_3_ group (0,ν_2_) (left panels), of the reaction coordinate CH_3_–I (ν_1_,0) (middle panels), or from the set including all of these modes (ν_1_,ν_2_) (right panels). We compare the spectrum obtained from the ground state (0,0), to those obtained using a passive control approach where the initial vibrational state that maximize the spectra is chosen at each energy out of the corresponding set of selected states, $\sigma_{max}^{e}\left(E\right)\left(e=I,I^{*}\right)$ , and to those obtained from the optimized initial superposition of selected states that maximizes the functional, $\sigma_{opt}^{e}\left(E\right)$ .

As expected from the photospectra (see Figure 1), σ_max_(*E*) coincides with σ_(0,0)_(*E*) at intermediate excitation energies, around 5 eV. For the I* fragment, $\sigma_{\max}^{I^{*}}\left(E\right)$ then increases at lower energies as one chooses an initial vibrational state with larger ν_1_, and becomes wider at higher energies as the starting state has larger ν_2_. Choosing among the 30 states from the (ν_1_,ν_2_) set allows for an effective widening of the $\sigma_{\max}^{I^{*}}\left(E\right)$ with respect to $\sigma_{(0,0)}^{I^{*}}\left(E\right)$ for a large set of energies. In fact, the decay of the photospectra at high energies is induced by our choice of limiting the set of vibrational states from which we choose the starting wave function, to those with ν_1_ ≤ 5 and ν_2_ ≤ 4. On the other hand, $\sigma_{\max}^{I}\left(E\right)$ highly increases with respect to $\sigma_{(0,0)}^{I}\left(E\right)$ at higher energies as one chooses states with larger ν_1_, while the effect of ν_2_ is quite small.

As the geometrical optimization is a variational scheme, the optimized spectra displays cross sections always larger than those of the maximum spectra, $\sigma_{\mathrm{opt}}^{e}\left(E\right)\geq \sigma_{\max}^{e}\left(E\right)$ . But the impact of the optimization depends crucially on the choice of the modes that create the superposition. As Figure 2 reveals, while the (0,ν_2_) set barely changes $\sigma_{opt}^{e}\left(E\right)$ from $\sigma_{\max}^{e}\left(E\right)$ , (ν_1_,0) makes $\sigma_{\mathrm{opt}}^{I^{*}}\left(E\right)$ more than twice larger than $\sigma_{\max}^{I^{*}}\left(E\right)$ except at very small or very large energies, where the spectra naturally tend to zero (although the blue-edge of the spectra is, as noted before, artificially truncated by limiting ν_1_ ≤ 5). In the case of $\sigma_{\mathrm{opt}}^{I}\left(E\right)$ , the optimization only improves the $\sigma_{\max}^{I}\left(E\right)$ values for *E* ≳ 5 eV. Finally, as expected from any variational method, the optimization using the initial vibrational eigenstates of both modes achieves the best results, but the improvement over the results using only the set of (ν_1_,0) states is not overwhelming.

The total output of the photodissociation reaction is proportional to the integral of the cross section, so the ratio of these integrals measures the gain in products that can be achieved by optimizing the wave function (for each excitation energy) using weak fields. We call it the gain over the best output choosing a single vibrational state,

10 $$g_{o|m}^{e}=\frac{\int_{0}^{\infty }\sigma_{\mathrm{opt}}^{e}\left(E\right)dE-\int_{0}^{\infty }\sigma_{\max}^{e}\left(E\right)dE}{\int_{0}^{\infty }\sigma_{\max}^{e}\left(E\right)dE}$$

and similarly we define the gain over the standard conditions (starting from the ground vibrational state) as $g_{o|0}^{e}=\left(\int \sigma_{\mathrm{opt}}^{e}\left(E\right)dE/\int \sigma_{(0,0)}^{e}\left(E\right)dE\right)-1$ . Using optimized superpositions of CH_3_ group modes the gains are modest: around $g_{o|m}^{e}∼0.1$ for both I and I* (a ∼ 10% increase), while $g_{o|0}^{I}=0.54$ and $g_{o:0}^{I^{*}}=0.94$ . But using the CH_3_–I stretching vibrational states we find gains $g_{o|m}^{e}$ over 100% for both products (or over 400% with respect to standard conditions): $g_{o|m}^{I}=1.18$ , $g_{o|m}^{I^{*}}=1.34$ , $g_{o|0}^{I}=4.44$ , $g_{o|0}^{I^{*}}=4.03$ . Finally, the integrated optimized spectra using all the modes is 54% larger than the integrated spectra using the CH_3_–I mode for the I channel, while it is 30% larger for the I* channel. The gain in the first case is $g_{o|m}^{I}=1.61$ (and a remarkable $g_{o|0}^{I}=7.39$ with respect to the ground state), but it is practically the same as the gain with the (ν_1_,0) set for the I* channel, $g_{o|m}^{I^{*}}=1.36$ (although the gain is large with respect to the ground state, $g_{o|0}^{I^{*}}=5.52$ ). This reflects the fact that using all modes, $\sigma_{\max}^{I^{*}}\left(E\right)$ increases quite more than $\sigma_{\max}^{I}\left(E\right)$ compared to the ground state spectra.

### Competition

As described in the Methods, in addition to maximizing each cross section independently, one can also maximize the total output of products, in our case, regardless of the chemical channel I or I*, by solving the eigenvalue equation **S** ^t^(*E*)**c**(*E*) = σ^t^(*E*)**c**(*E*) ( $\sigma_{\mathrm{opt}}^{t}\left(E\right)$ is the largest eigenvalue), where $S^{t}\left(E\right)=S^{I}\left(E\right)+S^{I^{*}}\left(E\right)$ . The results are shown in Figure 3A–C where the optimization is carried out using the (ν_1_,0), (0,ν_2_) and (ν_1_,ν_2_) sets of states, as previously. In the figure we also show the contribution of each species, $\sigma_{\mathrm{opt}}^{t,I}$ and $\sigma_{\mathrm{opt}}^{t,I^{*}}$ , to the total cross section. The results provide interesting information on how maximizing the same functional for both product channels (instead of maximizing a different functional for each channel, as done in Figure 2) affects each of the channels. As expected, I* is very dominant al low energies, while I is very dominant at high energies, except perhaps in the optimization with the (0,ν_2_) set. But again, the CH_3_ group modes barely allow any optimization, as $\sigma_{\mathrm{opt}}^{t}\left(E\right)\approx \sigma_{\max}^{t}\left(E\right)$ , while $\sigma_{\mathrm{opt}}^{t}\left(E\right)$ doubles $\sigma_{\max}^{t}\left(E\right)$ for most energies starting from a superposition of vibrational states of the reaction coordinate, and a similar result is achieved using the complete (ν_1_,ν_2_) set.

**Figure 3:**
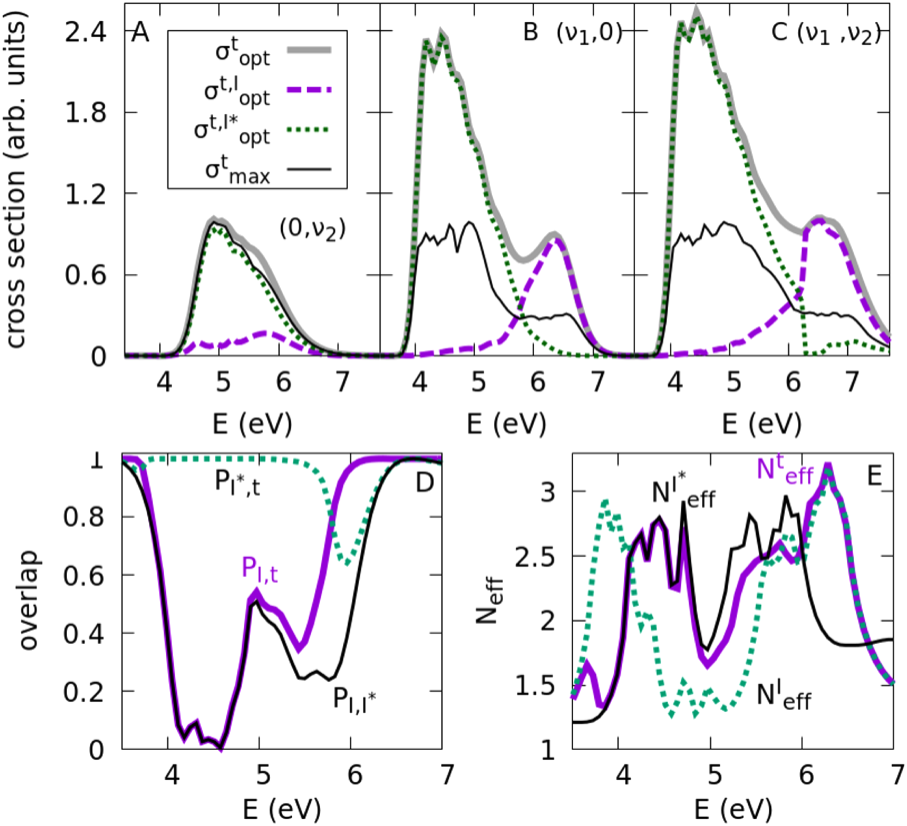
(A) Optimized total cross sections of photodissociation that maximize the functional *F* ^*t*^, using excited vibrational modes of the CH_3_ group (0,ν_2_), (B) the reaction coordinate CH_3_–I (ν_1_,0), (C) or the set including all of these modes (ν_1_,ν_2_). We show the contribution of each channel, $\sigma_{\mathrm{opt}}^{t,I}\left(E\right)$ and $\sigma_{\mathrm{opt}}^{t,I^{*}}\left(E\right)$ to the total cross section, $\sigma_{\mathrm{opt}}^{t}\left(E\right)$ , as well as the maximun total cross section obtained starting from a single vibrational state within the selected set of states, $\sigma_{\max}^{t}\left(E\right)$ . (D) Square of the overlap of the optimized wave functions, $P_{I^{*},t}\left(E\right)=\left|\left\langle \psi_{\mathrm{opt}}^{I^{*}}\left(E\right)\right|\psi_{\mathrm{opt}}^{t}\left(E\right)\right\rangle {|}^{2}$ , $P_{I,t}\left(E\right)=\left|\left\langle \psi_{\mathrm{opt}}^{I}\left(E\right)\right|\psi_{\mathrm{opt}}^{t}\left(E\right)\right\rangle {|}^{2}$ and $P_{I,I^{*}}\left(E\right)=\left|\left\langle \psi_{\mathrm{opt}}^{I}\left(E\right)\right|\psi_{\mathrm{opt}}^{I^{*}}\left(E\right)\right\rangle {|}^{2}$ , obtained using the (ν_1_,0) set of states, that measures the compatibility between the different targets. (E) Effective number of states that contribute to the optimized superpositions using the (ν_1_,0) set, $N_{\mathrm{eff}}^{e}\left(E\right)\left(e=I,I^{*},t\right)$ , which measures the importance of the constructive interference in the optimization, as described in the text.

The gains in total cross sections are similar to those obtained previously in the maximization of I and I* independently, e.g., $g_{o|m}^{t}=1.27,0.081,1.35$ using the (ν_1_,0), (0,ν_2_), (ν_1_,ν_2_) sets, respectively. The efficiency of the maximization of all the products can be estimated by comparing the total cross sections to those of the independent product optimizations. We define the efficiency parameter

11 $$\gamma =\int_{0}^{\infty }dE\frac{\sigma_{\mathrm{opt}}^{t}\left(E\right)}{\sigma_{\mathrm{opt}}^{I}\left(E\right)+\sigma_{\mathrm{opt}}^{I^{*}}\left(E\right)}$$

If **S** ^I^(*E*) and $S^{I^{*}}\left(E\right)$ would commute, they would share the same eigenstates, such that the maximization of both products would be compatible (it could be obtained starting from the same optimized superposition state) and γ = 1. With the (ν_1_,0) set we obtain γ = 0.93, with (0,ν_2_) we get γ = 0.95, and with the complete set we obtain γ = 0.89. For the most dominant product, I*, the efficiency [measured as $\int_{0}^{\infty }dE\sigma_{\mathrm{opt}}^{t,I^{*}}\left(E\right)/\int_{0}^{\infty }dE\sigma_{\mathrm{opt}}^{I^{*}}\left(E\right)$ ] is always large and close to 0.95, but one can still achieve an efficiency near 0.80 for the I product [measured as $\int_{0}^{\infty }dE\sigma_{\mathrm{opt}}^{t,I}\left(E\right)/\int_{0}^{\infty }dE\sigma_{\mathrm{opt}}^{I}\left(E\right)$ ], implying that the maximization of the total amount of products is not obtained at the expense of only one of them. Still, the contributions to the total cross section from the I and I* channels in Figure 3B,C occur mainly at different energies, so the bands barely overlap. Therefore, at each energy, the optimization mainly occurs by maximizing constructive interference for the product that is dominant. In Figure 3D we show the square of the overlap between the optimized wave functions that maximize the spectra using the (ν_1_,0) set, as an example. The wave function that maximizes the total spectra greatly overlaps with the wave function that maximizes the output of I* at low and middle energies, $\left(P_{I^{*},t}\left(E\right)\equiv |\langle \psi_{\mathrm{opt}}^{I^{*}}\left(E\right)\left|\psi_{\mathrm{opt}}^{t}\left(E\right)\right\rangle {|}^{2}\right)$ , and with the wave function that maximizes the output of I at higher energies $\left(P_{I,t}\left(E\right)\equiv |\langle \psi_{\mathrm{opt}}^{I}\left(E\right)\left|\psi_{\mathrm{opt}}^{t}\left(E\right)\right\rangle {|}^{2}\right)$ , while for most energies (except at the fringes of the spectra) $P_{I,I^{*}}\left(E\right)\equiv \left|\left\langle \psi_{\mathrm{opt}}^{I}\left(E\right)\right|\psi_{\mathrm{opt}}^{I^{*}}\left(E\right)\right\rangle {|}^{2}$ is very small. However, there is some degree of overlap (compatibility) between the optimal wave functions around ∼5 eV that becomes very high at energies ≳6 eV.

As discussed in the Methods, the cross section from an initial superposition state depends on the matrix elements of the scattering matrix **S** ^*e*^(*E*) (*e* = *I*, I*, *t*), and as long as the matrix has nondiagonal elements, the spectra will exhibit interference patterns. The optimization rests upon maximizing the constructive interference on the desired channel. Expanding eq 6 in its diagonal and off-diagonal contributions, we obtain

σopte=∑jN|cj|2Sjje+∑jN∑k>jN2Re[cj*ckSjke]=σdiage+σodiae 12

where the subscripts refer to the vibrational states with which the spectra is obtained, and Re is the real part of the complex number. The cross section is the sum of the diagonal $\sigma_{\mathrm{diag}}^{e}$ and the off-diagonal $\sigma_{\mathrm{odia}}^{e}$ contributions Here and in the following discussion we omit the energy dependence of all quantities, $\sigma_{opt}^{e}\left(E\right)$ , *c*_*j*_(*E*) and $S_{jk}^{e}\left(E\right)$ , for brevity. It is $\sigma_{\mathrm{odia}}^{e}$ , which depends on the off-diagonal matrix elements of **S** ^*e*^ and is controlled by the initial vibrational coherences (the products $c_{j}^{*}c_{k}$ ), that is responsible for the coherent control. The maximal effect of the coherences is limited by the number of participating states. It can be easily proven that the ratio $\sigma_{\mathrm{opt}}^{e}/\sigma_{\max}^{e}\leq N$ . If we call $S_{\max}^{e}\equiv \sigma_{\max}^{e}$ , the largest cross section that can be obtained from the set of vibrational states considered, *j* = *v*_max_, then $S_{jk}^{e}\leq S_{\mathrm{opt}}^{e}$ and $\sigma_{\mathrm{opt}}^{e}\leq S_{\max}^{e}\overset{N}{\underset{j}{\sum }}\overset{N}{\underset{k}{\sum }}c_{j}^{*}c_{k}$ . The last term is convex on the coefficients, so it is maximal when all the coefficients are equal, $c_{j}=1/\sqrt{N}$ , forming the fully symmetrical superposition. This superposition is equivalent to the state responsible for parallel transfer^34,41^ in the context of ultrafast absorption, or for strong-coupling in cavity quantum electrodynamics. Then

13 $$1\leq \frac{\sigma_{\mathrm{opt}}^{e}}{\sigma_{\max}^{e}}\leq \overset{N}{\underset{j}{\sum }}\overset{N}{\underset{k}{\sum }}\frac{1}{N}=N$$

We define the effective number of participating states, $N_{\mathrm{eff}}^{e}\left(E\right)\equiv \sigma_{\mathrm{opt}}^{e}\left(E\right)/\sigma_{\max}^{e}\left(E\right)$ as the ratio of the spectra at each energy. Although in general, all *N* states participate in the optimized superposition of the spectra (some with vanishingly small contributions), $N_{\mathrm{eff}}^{e}\left(E\right)$ gives the number of states that would form an ideal superposition where maximal constructive interference could be achieved if all $\sigma_{v}^{e}\left(E\right)$ would be identical, such that the optimization gives the same final results. As such, it quantifies the importance of the constructive interference in the control.

In Figure 3E, the effective number of states is shown for the optimizations of $\sigma_{\mathrm{opt}}^{I}\left(E\right)$ , $\sigma_{\mathrm{opt}}^{I^{*}}\left(E\right)$ and $\sigma_{\mathrm{opt}}^{t}\left(E\right)$ for the (ν_1_,0) set. $N_{\mathrm{eff}}^{e}\left(E\right)$ is typically bounded between 1.5 and 3, with larger values whenever $\sigma_{opt}^{e}\left(E\right)$ is large: at lower energies for $N_{\mathrm{eff}}^{I^{*}}\left(E\right)$ and at higher energies for $N_{\mathrm{eff}}^{I}\left(E\right)$ , while it remains large for $N_{\mathrm{eff}}^{t}\left(E\right)$ across all the energy range, except around ∼5 eV, where there is a small dip in $N_{\mathrm{eff}}^{T}\left(E\right)$ (and in fact in all $N_{\mathrm{eff}}^{e}\left(E\right)$ ).

A more striking competition between different tasks is embodied in the functional that maximizes the difference spectrum $\sigma^{d}\left(E\right)=\sigma^{I}\left(E\right)-\sigma^{I^{*}}\left(E\right)$ , which involves maximizing one species and minimizing the other. In the more general sense, difference spectra are constructed to contrast signals such that small but important factors can be more easily identified (e.g., in circular dichroism). However, when the separation between the signals is very small, features of the difference spectra can be noise-induced ghost signals. Optimization techniques can be used as diagnostics to discriminate physical or relevant signals (they should raise if the process is optimized) from unphysical ones. In the context of this work, the difference spectra implies selecting one product (the least dominant) over the other one, but in a way where the amount of the selected product (the signal) is important. So it strikes a balance between the efficiency and the selectivity in the reaction.

In Figure 4 we show the optimized difference spectra obtained with the (ν_1_,0),(0,ν_2_) and (ν_1_,ν_2_) sets, together with the maximum difference spectra that can be achieved by initiating the system in a single vibrational eigenstate of the same set. We also show the contribution from each product species, $\sigma^{d,I}$ ­(*E*) and σ^d,I*^(*E*), for both the optimized and maximal spectra (the σ^d,I*^ is shown in the negative half of the ordinate axis, as this contribution is subtracted in σ^*d*^). For the (0,ν_2_) set, the optimization barely improves the maximal spectrum: as in the previous total cross sections, superpositions do not lead to significant interference. Hence $\sigma_{\mathrm{opt}}^{d}\left(E\right)$ is negative at lower energies (where I* dominates) and positive at higher energies (where I dominates).

**Figure 4:**
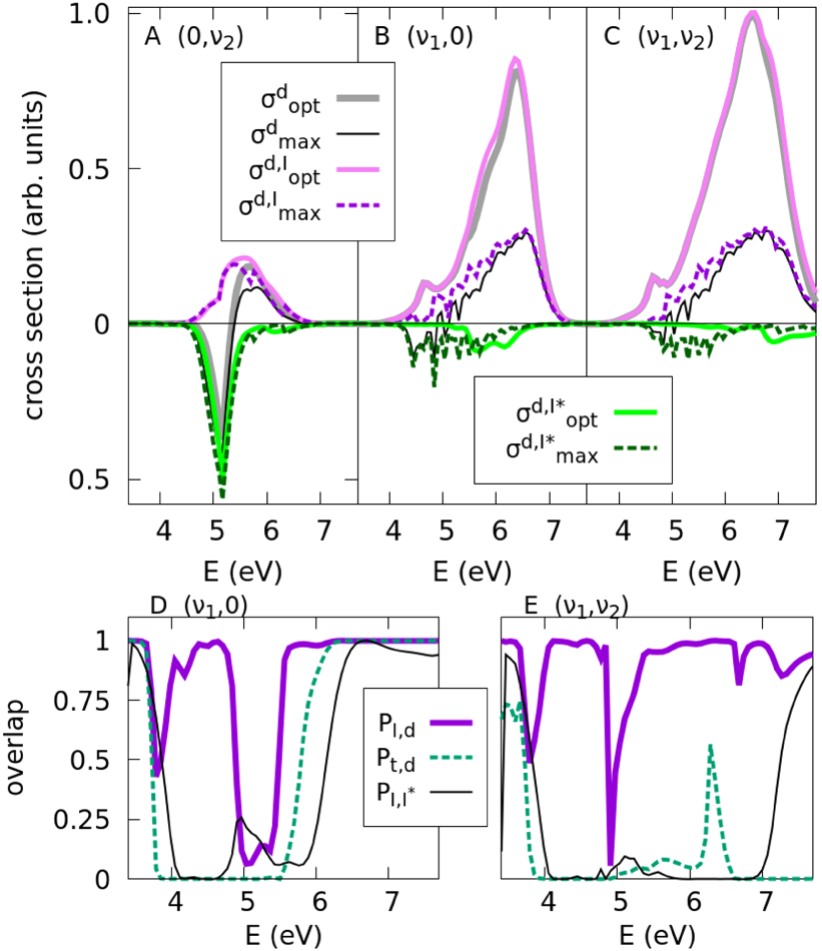
(A)–(C) Difference spectra obtained with the optimized superposition that maximizes the functional $F^{d}=\sigma_{\mathrm{opt}}^{d}\left(E\right)$ , using the sets of (0,ν_2_), (ν_1_,0) and (ν_1_,ν_2_) vibrational states, and with the selected vibrational eigenstate within these sets that maximizes the difference spectra, $\sigma_{\max}^{d}\left(E\right)$ . Also shown are the contributions to the cross section from the I channel $\sigma^{d,I}$ (positive axis), and the I* channel $\sigma^{d,I^{*}}$ (negative axis). The square of the overlap between the optimized wave functions, $P_{I,d}\left(E\right)=\left|\left\langle \psi_{\mathrm{opt}}^{I}\left(E\right)\right|\psi_{\mathrm{opt}}^{d}\left(E\right)\right\rangle {|}^{2}$ , $P_{t,d}\left(E\right)=\left|\left\langle \psi_{\mathrm{opt}}^{t}\left(E\right)\right|\psi_{\mathrm{opt}}^{d}\left(E\right)\right\rangle {|}^{2}$ and $P_{I,I^{*}}\left(E\right)=\left|\left\langle \psi_{\mathrm{opt}}^{I}\left(E\right)\right|\psi_{\mathrm{opt}}^{I^{*}}\left(E\right)\right\rangle {|}^{2}$ , is shown for the results obtained using the (ν_1_,0) (D) and (ν_1_,ν_2_) (E) sets of states.

On the other hand, both with the (ν_1_,0) and (ν_1_,ν_2_) sets, $\sigma_{\mathrm{opt}}^{d}\left(E\right)$ is positive for all energies, as $\sigma_{\mathrm{opt}}^{d}\left(E\right)$ almost coincides with $\sigma_{\mathrm{opt}}^{d,I}\left(E\right)$ , since $\sigma_{\mathrm{opt}}^{d,I^{*}}\left(E\right)$ is practically quenched. Actually, for these two sets of vibrational states (and also for the (0,ν_2_) set), $\sigma_{\mathrm{opt}}^{d,I^{*}}\left(E\right)<\sigma_{\max}^{d,I^{*}}\left(E\right)$ across most of the excitation energy range. Except at low energies, this is also the case for $\sigma_{\max}^{d}\left(E\right)$ , and $\sigma_{\max}^{d,I}\left(E\right)$ . Comparing $\sigma_{\mathrm{opt}}^{d,I}\left(E\right)$ with $\sigma_{\max}^{d,I}\left(E\right)$ , we observe that the cross sections have a similar shape (except at low energies) but the amplitude in the optimal cross section is twice or more than twice $\sigma_{\max}^{d,I}\left(E\right)$ . In fact, $\sigma_{\mathrm{opt}}^{d,I}\left(E\right)$ are very similar to $\sigma_{\mathrm{opt}}^{I}\left(E\right)$ of Figure 2 for both (ν_1_,0) and (ν_1_,ν_2_) sets, which implies that one can suppress the dominant product I* without barely affecting the output of I products. Thus, a functional like $F^{d}=\sigma^{d}\left(E\right)=\sigma^{I}\left(E\right)-\sigma^{I^{*}}\left(E\right)$ appears to be able to maximize the output of I products at a similar level as the *F* ^*I*^ = σ^*I*^(*E*) functional does in Figure 2, through constructive interference, while simultaneously quenching the production of I fragments through destructive interference.

The previous discussion suggests that the optimized wave functions that maximize $\sigma_{\mathrm{opt}}^{d}\left(E\right)$ and $\sigma_{\mathrm{opt}}^{I}\left(E\right)$ , namely $\psi_{\mathrm{opt}}^{d}\left(E\right)$ and $\psi_{\mathrm{opt}}^{I}\left(E\right)$ , are rather similar. In Figure 4D and E we show the overlaps $P_{I,d}\left(E\right)\equiv \left|\left\langle \psi_{\mathrm{opt}}^{I}\left(E\right)\right|\psi_{\mathrm{opt}}^{d}\left(E\right)\right\rangle {|}^{2}$ , $P_{t,d}\left(E\right)\equiv \left|\left\langle \psi_{\mathrm{opt}}^{t}\left(E\right)\right|\psi_{\mathrm{opt}}^{d}\left(E\right)\right\rangle {|}^{2}$ and $P_{I,I^{*}}\left(E\right)$ for (ν_1_,0) and (ν_1_,ν_2_). While $\psi_{\mathrm{opt}}^{d}\left(E\right)$ is similar to $\psi_{\mathrm{opt}}^{I}\left(E\right)$ for most of the spectra except at specific energies around 3.8 and 5 eV (the overlap is close to 1), $\psi_{\mathrm{opt}}^{d}\left(E\right)$ is very different from $\psi_{\mathrm{opt}}^{t}\left(E\right)$ . In spite of using more states, the overlap $P_{I,d}\left(E\right)$ is even larger using the (ν_1_,ν_2_) set. This can be explained from the fact that the overlap between $\psi_{\mathrm{opt}}^{I}\left(E\right)$ and $\psi_{\mathrm{opt}}^{I^{*}}\left(E\right)$ is smaller using all the states. Then, maximizing I is clearly (to say the least) not affecting $\sigma^{I^{*}}\left(E\right)$ .

Following our previous discussion, we can define the gain of products in channel I achieved during the optimization of $\sigma_{\mathrm{opt}}^{d}\left(E\right)$ as

14 $$g_{o|m}^{d,I}=\frac{\int_{0}^{\infty }\sigma_{\mathrm{opt}}^{d,I}\left(E\right)dE-\int_{0}^{\infty }\sigma_{\max}^{d,I}\left(E\right)dE}{\int_{0}^{\infty }\sigma_{\max}^{d,I}\left(E\right)dE}$$

As noticed, this gain is obtained by constructive interference induced by the initial vibrational coherences. The results give $g_{o|m}^{d,I}=0.16,1.40,1.86$ for the (0,ν_2_), (ν_1_,0) and (ν_1_,ν_2_) sets, respectively. These gains are better than those found in the optimization of $\sigma_{\mathrm{opt}}^{I}\left(E\right)$ or $\sigma_{\mathrm{opt}}^{t}\left(E\right)$ . Interestingly, using the complete set of vibrational states provides a substantial gain over the (ν_1_,0) set.

We can similarly quantify the destructive interference induced over channel I* by the initial vibrational coherences by defining the loss of product I* achieved during the optimization of $\sigma_{\mathrm{opt}}^{d}\left(E\right)$ as

15 $$l_{o|m}^{d,I^{*}}=\frac{\int_{0}^{\infty }\sigma_{\max}^{d,I^{*}}\left(E\right)dE-\int_{0}^{\infty }\sigma_{\mathrm{opt}}^{d,I^{*}}\left(E\right)dE}{\int_{0}^{\infty }\sigma_{\max}^{d,I^{*}}\left(E\right)dE}$$

We obtain values of $l_{o|m}^{d,I^{*}}=0.26,0.43,0.46$ for the (0,ν_2_), (ν_1_,0) and (ν_1_,ν_2_) sets, respectively. There is actually some suppression (around ∼26%) of the I* using a superposition of CH_3_ group modes over the maximal cross section obtained with a single eigenstate, but the effect is clearly more pronounced using a superposition of the CH_3_–I stretching, or all the modes. Unlike in the gain of the I products, using all the modes barely changes the loss of the I* product with respect to using the (ν_1_,0) set.

### Discrimination

Now we will discuss the results when we maximize the quantum yield of the least dominant channel, I, that we obtain from the optimized superposition that gives the largest eigenvalue solving the nonlinear eq 7. That is, we solve the eigenvalue equation for the functional constructed to maximize the yield. In Figure 5 (upper panels) we show the results using the three different sets of states (0,ν_2_), (ν_1_,0) and (ν_1_,ν_2_) comparing, as before, the optimized yield $\chi_{\mathrm{opt}}^{I}\left(E\right)$ , the maximal yield that can be achieved by starting from a single vibrational eigenstate (the so-called passive control conditions), $\chi_{\max}^{I}\left(E\right)$ , and the yield in normal conditions $\chi_{(0,0)}^{I}\left(E\right)$ , obtained by starting from the ground state. The interesting part of the spectra occurs in the region between 4–6 eV, corresponding to the A band in normal conditions, where I* dominates. In the lower panels we show the cross sections for the I fragments (the desired output) obtained when we start from the optimized superposition $\sigma_{\mathrm{opt}}^{\chi ,I}\left(E\right)$ , the selected eigenstate that maximizes the yield $\sigma_{\max}^{\chi ,I}\left(E\right)$ and the ground state $\sigma_{(0,0)}^{I}\left(E\right)$ .

**Figure 5:**
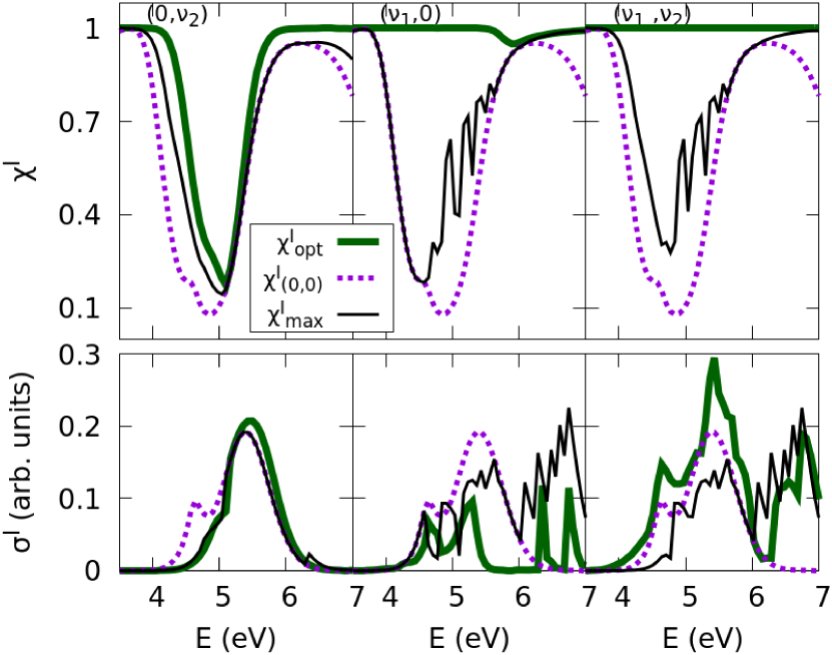
(Upper panels). Quantum yields of the I fragment in the A band obtained from the optimized superposition states that maximize the yield $\chi_{\mathrm{opt}}^{I}$ using vibrational states of the CH_3_ group modes (0,ν_2_), the CH_3_–I stretching (ν_1_,0) and both modes (ν_1_,ν_2_). For comparison we show as well the quantum yields obtained starting from the ground state $\chi_{(0,0)}^{I}$ and from the single eigenstate selected from the corresponding set of vibrational states that maximizes the yield, $\chi_{\max}^{I}$ . (Lower panels). Cross sections on the I fragment obtained starting from the optimized wave function, the ground state and the eigenstate leading to $\chi_{\max}^{I}$ .

Interestingly, comparing $\chi_{\max}^{I}\left(E\right)$ with $\chi_{(0,0)}^{I}\left(E\right)$ in all panels, we observe that starting from a single vibrational eigenstate does not improve much the yield over the ground state. It is really necessary to use optimized superposition states. Again, when using CH_3_ group modes the result of the optimization only shows a small improvement over the uncontrolled ones. However, the optimized superposition state using the 6 lowest vibrational states of the CH_3_–I stretching mode can practically guarantee a perfect yield ∼1 across the whole energy range of the I channel, although this comes at the expense of quenching the cross section, particularly between 5.5–6 eV. This shows that the control is mainly exerted by destructive interference over the I* channel: although $\sigma_{\mathrm{opt}}^{\chi ,I}\left(E\right)$ is much smaller than $\sigma_{\max}^{\chi ,I}\left(E\right)$ at some energies, $\sigma_{\mathrm{opt}}^{\chi ,I^{*}}\left(E\right)$ (not shown) is practically quenched, so that $\chi_{\mathrm{opt}}^{I}∼1$ (except near 6 eV). Using the full set of vibrational states, on the other hand, allows perfect selectivity ( $\chi_{\mathrm{opt}}^{I}\left(E\right)=1$ for all energies) *and* this is not achieved at the expense of quenching $\sigma_{\mathrm{opt}}^{\chi ,I}\left(E\right)$ , which is of the order of or larger than $\sigma_{\max}^{\chi ,I}\left(E\right)$ . While the selectivity is achieved mainly due to destructive interference on the undesired channel, when using all the states one can still observe the effect of constructive interference over the desired channel.

We have studied the results of geometrical optimization applied to different goals, obtaining a larger amount of products, a larger amount of one product versus the other, or maximizing the quantum yield, for which we set up different functionals. Some of the functionals could in principle imply both efficiency in gaining more amount of products and selectivity. In Figure 6 we compare the selectivity achieved measured with the branching ratio, $\sigma^{I}/\sigma^{I^{*}}$ , which is most sensitive to the quenching of the undesired channel, and we show the results in logarithmic scale. We choose to compare the results obtained by maximizing the functionals *F* ^*I*^ (which maximizes σ^*I*^(*E*)), *F* ^*d*^ (which maximizes σ^*d*^(*E*)) and *F* ^χ^ (which maximizes $\chi^{I}\left(E\right)$ ). Indeed we could have also constructed a functional depending directly on the branching ratio, although minimizing the quantity $c^{T}S^{I^{*}}c$ in the denominator makes the nonlinear eigenvalue equation sometimes more difficult to converge. The results (not shown) are exactly the same as those obtained using *F* ^χ^, except for the very few energies where convergence is not achieved. In addition, we show the branching ratio starting from the ground state (0, 0), and from the single eigenstate that maximizes the branching ratio (or quantum yield), out of the sets of (ν_1_,0) and (ν_1_,ν_2_) states.

**Figure 6:**
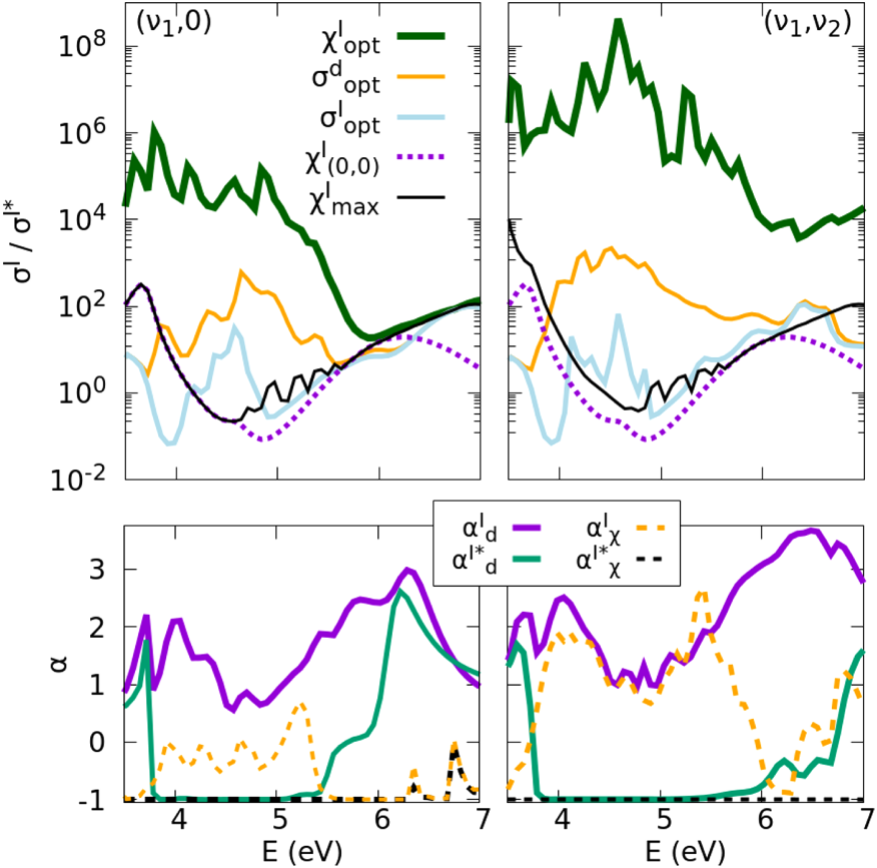
(Upper panels) Branching ratio $\sigma^{I}/\sigma^{I^{*}}$ between the two product channels shown in logarithmic scale vs energy. The different curves are obtained when the photodissociation starts from the wave function that maximizes the quantum yield $\chi_{\mathrm{opt}}^{I}$ , the output of the I product $\sigma_{\mathrm{opt}}^{I}$ , the difference spectrum σ_opt_^d^, the ground state, $\chi_{(0,0)}^{I}$ and the single eigenstate that maximizes the quantum yield $\chi_{\max}^{I}$ chosen within the set of vibrational states (ν_1_,0) (left panel) or (ν_1_,ν_2_) (right panel). (Lower panels) Coherence parameter for the I and I* channels, evaluated for the functionals *F* ^*d*^ and *F* ^χ^, $\alpha_{d}^{e}$ and $\alpha_{\chi }^{e}\left(e=I,I^{*}\right)$ , respectively. The coherence parameter, positive for constructive interference and negative for destructive interference (see the text), is calculated when the optimization is performed with the (ν_1_,0) (left panel) and the (ν_1_,ν_2_) (right panel) set of states.

The results of Figure 6 show that the curve that maximizes the branching ratio using a single eigenstate behaves very much like the branching ratio for the ground state. The selectivity is larger for $\chi_{\max}^{I}$ (notice that the y-scale is logarithmic) but the unwanted product is not fully discriminated. The same happens with the results obtained with *F* ^*I*^ and *F* ^*d*^. The latter always improves the selectivity. The shape of the curves is similar for the (ν_1_,0) set, not so much for the (ν_1_,ν_2_) set. In the regions of the spectra where I* naturally dominates, using the superposition that maximizes the difference spectra, the output of I* can be smaller than a 0.1% of that of *I*. However, maximizing the quantum yield really leads to maximal use of destructive interference over the I* channel, such that for most of the A band, the branching ratio of I*/I products is less than one particle in a million. Thus, what we may call “discrimination functionals″ lead to a very high product channel selectivity, maximizing destructive interference on the unwanted channel. We can quantify the contribution of the different terms in the interference by defining the *coherence parameter*, $\alpha_{F}^{e}\left(E\right)$ ,

16 $$\alpha_{F}^{e}\left(E\right)=\frac{\sigma_{\mathrm{diag}}^{F,e}\left(E\right)-\sigma_{\mathrm{odia}}^{F,e}\left(E\right)}{\sigma_{\mathrm{diag}}^{F,e}\left(E\right)}$$

where *e* = *I*, I* and the functional, *F* ^*d*^ or *F* ^*χ*^, will be indicated as *F* = *d*, *χ*. Because the cross sections are always positive, for the channel that is suppressed (I*), the absolute value of the off-diagonal term can be at most as large as the diagonal term, but with negative sign, for complete destructive interference. Then $\alpha_{F}^{e}\left(E\right)$ is bounded from below, with a minimum of $\alpha_{F}^{e}\left(E\right)=-1$ . Negative values of $\alpha_{F}^{e}\left(E\right)$ always indicate that the interference is destructive, while positive values indicate that there is constructive interference. But there is no upper bound for $\alpha_{F}^{e}\left(E\right)$ as $\sigma_{\mathrm{diag}}^{e,F}\left(E\right)$ could be zero. We have found that, for typical cases where the cross section is dominated by one (or few) vibrational contributions at a particular energy, $\alpha_{F}^{e}\left(E\right)∼N_{\mathrm{eff}}^{e}\left(E\right)-1$ for the product being selected (the I channel, in our case).

In the lower panels of Figure 6 we show the coherence parameter for the I and I* channels, evaluated for the functionals *F* ^*d*^ and *F* ^χ^, using the (ν_1_,0) (bottom left) and the (ν_1_,ν_2_) (bottom right) sets of vibrational states. For the *F* ^*d*^ functional using the (ν_1_,0) set, we observe constructive interference for the cross section $\sigma_{\mathrm{opt}}^{d,I}\left(E\right)$ across all energies, inducing $\alpha_{d}^{I}\left(E\right)>0$ . Destructive interference is important and almost perfect only for the energies where the I* channel is more predominant (*E* ∼ [4,5] eV). Using the full set of states the results are similar, with larger positive values of $\alpha_{d}^{I}\left(E\right)$ and negative values of $\alpha_{d}^{I^{*}}\left(E\right)$ along a larger energy range.

On the other hand, for the *F* ^χ^ functional using the (ν_1_,0) set, the destructive interference is almost complete for the I* channel across all the energy range considered, $\alpha_{\chi }^{I^{*}}\left(E\right)∼-1$ , while $\alpha_{\chi }^{I}\left(E\right)$ is mostly negative! To maximize the discrimination it is not necessary to increase the cross section of the selected channel, $\sigma_{\mathrm{opt}}^{\chi ,I}\left(E\right)$ , as long as the unwanted channel is totally suppressed, $\sigma_{\mathrm{opt}}^{\chi ,I^{*}}\left(E\right)\approx 0$ . This could be inferred inspecting the cross sections in Figure 5. Only when we perform the optimization using the full set of (ν_1_,ν_2_) vibrational states, one can use constructive interference on the I channel $\left(\alpha_{\chi }^{I}\left(E\right)>0\right)$ while maintaining the complete destructive interference over the I* channel for most energies across the A band. While for other observables the results using the full set of states typically did not improve greatly over the results using only the CH_3_–I modes, the discrimination of the unwanted species really benefits from using a larger basis, such that the branching ratio with the full set is over 100 times larger than with the (ν_1_,0) set. Interestingly, this remarkable selectivity is mostly observed at lower energies, whereas the selectivity is smaller (albeit very good) at higher energies, where I naturally dominates. But this result is an artifact of the cutoff in the basis, which does not allow to fully use the coherence-induced interference at large energies.

From the above results it becomes clear the importance of designing the functional to be maximized in order to achieve the maximum degree of control on the magnitude of interest. Therefore, it is interesting to discuss now how different functionals can be implemented in a practical experimental application of the present control scheme. As discussed earlier,^45^ a typical experimental implementation of this control scheme would apply a strong infrared (IR) pulse or a two pulse sequence, to prepare the initial superposition of vibrational states in the ground state potential, by multiphoton IR absorption or stimulated Raman transitions. The vibrational superposition could be optimized by means of pulse shaping, applying an adaptive feedback genetic algorithm. In a pump–probe photodissociation experiment, the σ^*I*^ and $\sigma^{I^{*}}$ cross sections of the two fragment channels are routinely measured. This is the only information required to design the desired functional to be introduced in the adaptive feedback genetic algorithm.

## Conclusions

We have derived and applied the geometrical optimization methodology to modify the photodissociation spectra of CH_3_I in the A band. Preparing optimized initial wave functions that maximally exploit interference-induced coherent control, we have shown that it is possible to increase the efficiency and selectivity of the photodissociation reaction using transform-limited ultrashort weak fields, even though the dynamics is mediated by uncontrolled nonadiabatic couplings.

To maximize the efficiency or selectivity of the reaction, it is necessary to design the proper functionals. We have shown results that use functionals that maximize the output of each product, of both products, or that imply competition between the products or selective discrimination of one of them, testing the performance of the methods and the effect of preparing initial vibrational coherences among CH_3_–I states, CH_3_ group states or both. We have defined different magnitudes that allow us to measure the contribution of constructive and destructive interference in the dynamics that lead to maximizing the different functionals. Our results showed that even in the weak field limit, it is possible to increase the efficiency of the reaction on any of the products by 100–200%, but only if the initial superposition at least uses vibrational states related to the CH_3_–I reaction coordinate. Analysis of the control showcases the utmost importance of constructive interference. It is also possible to increase the output of the least dominant product by almost the same amount (∼100%) and at the same time practically completely quench the output of products in the dominant channel. We have shown that this control requires both constructive interference in the desired channel and destructive interference in unwanted products. Finally, if one demands high product selectivity in the reaction, we showed that it is possible to design functionals able to suppress even the most dominant channel to less than one part in a million. In this case, maximizing the destructive interference on the undesired channel plays the major role. To avoid quenching of the signal and to reach even higher selectivity, we showed that it is important to prepare superposition states that involve all the available vibrational states, including those unrelated to the reaction coordinate.

The experimental implementation of different functionals should be straightforward in the framework of an adaptive feedback genetic algorithm, using the measured cross sections of the different fragment channels. The present conclusions appear to be general regarding the application of the control scheme to other photolysis reactions in polyatomic molecules that occur through different dissociation channels.

## Supplementary Material



## Notes

Details of the theoretical methodology applied in the simulations (PDF)
