# Supplementary material for: Preparing Superposition States to Modify the Spectra and to Achieve Complete Selectivity in Photodissociation Reactions
Source: J Chem Theory Comput. 2025 Jul 30;21(15):7267–78. doi: 10.1021/acs.jctc.5c00655 (PMC12355701; doi:10.1021/acs.jctc.5c00655)
Supplement: Supplementary file 1 [file ct5c00655_si_001.pdf]

SUPPORTING INFORMATION

Preparing superposition states to modify the spectra and to achieve complete selectivity in photodissociation reactions

Ignacio R. Sola[1] and Alberto García-Vela[2]

[1]Departamento de Química Física, Universidad Complutense de Madrid (and Unidad Asociada I+D+i CSIC), 28040 Madrid, Spain

[2]Instituto de Física Fundamental, Consejo Superior de Investigaciones Científicas, Serrano, 123, 28006 Madrid, Spain

e-mail: isolarei@ucm.es, garciavela@iff.csic.es

Theoretical model applied to describe the photodissociation of CH_3I

The methyl iodide system is represented by means of the reduced dimensionality model of Guo.[1,2] This model considers the CH_3I system as a CXI pseudotriatomic molecule where the pseudoatom X (X=H_3) is located at the center of mass of the three H atoms. In the model, three degrees of freedom represented by the (R, r, θ) Jacobi coordinates are included, where the dissociation coordinate R is the distance between I and the CH_3 (or C−X) center of mass, r is the C−X distance and it represents the umbrella bend of the C−H_3 group (ν_2), and θ is the angle between the vectors associated with R and r and it represents the X−C−I bend (ν_6). The assumption of modeling the umbrella mode of the C−H_3 group in CH_3I as a C−X stretch has been justified by a number of theoretical works, including both three[1−3] and two[4−6] dynamical degrees of freedom, which were successful in reproducing most

of the experimental data.

In the simulations zero total angular momentum, $J = 0$, is assumed for the system, and then the nuclear kinetic-energy operator can be expressed as

$$\hat{T} = -\frac{\hbar^2}{2\mu}\frac{\partial^2}{\partial R^2} - \frac{\hbar^2}{2m}\frac{\partial^2}{\partial r^2} + \left(\frac{1}{2\mu R^2} + \frac{1}{2I_{CH_3}}\right)\hat{j}^2, \quad (1)$$

where the reduced masses for CH_3I are defined as

$$\mu = \frac{m_I(m_C + m_{H_3})}{m_I + m_C + m_{H_3}} \;,\; m = \frac{m_C m_{H_3}}{m_C + m_{H_3}}, \quad (2)$$

where m_I, m_C, and m_{H_3} are the masses of the I, and C atoms, and of the three H atoms, respectively. In the present model the moment of inertia I_{CH_3} is assumed to be only the perpendicular component of the total moment of inertia,[1,7]

$$I_{CH_3} = m_H r_e^2(1 - \cos\gamma) + \frac{m_H m_C}{3m_H + m_C} r_e^2(1 + 2\cos\gamma), \quad (3)$$

being the H−C−H angle γ related to r through the relationship

$$\cos\gamma = 1.0 - 1.5\left[1.0 - \left(\frac{r}{r_e}\right)^2\right], \quad (4)$$

where it is assumed that the C_{3v} symmetry is preserved for CH_3. The C−H distance r_e has been fixed in our simulations to the value $r_e = 2.06573\ a_0$ in the case of the ground electronic state of the system, and to the value $r_e = 2.05266\ a_0$ in the case of the ${}^3Q_0$, ${}^1Q_1$, and ${}^3Q_1$ excited electronic states. The value $r_e = 2.06573\ a_0$ corresponds to the C−H distance in the equilibrium geometry of the ground state of CH_3I with C_{3v} symmetry found by *ab initio* calculations,[8] while $r_e = 2.05266\ a_0$ is the C−H distance found by the same calculations for the CH_3 equilibrium geometry with D_{3h} symmetry at very large CH_3−I separations.

Potential-energy surfaces and initial state

Photodissociation of CH_3I in the A band takes place upon excitation of the system from the $\tilde{X}^1A_1$ ground electronic state to the $^3Q_0$ excited electronic state (correlating with the CH_3 + I*($^2P_{1/2}$) products), which in turn is nonadiabatically coupled by a conical intersection to the $^1Q_1$ excited state (correlating with the CH_3 + I($^2P_{3/2}$) products). The $^3Q_1$ excited state (correlating also with the CH_3 + I($^2P_{3/2}$) products) can be radiatively accessed from the ground state, as well as the $^1Q_1$ state. Thus, four electronic potential-energy surfaces are involved in the photolysis process. The $\tilde{X}^1A_1$ ground electronic potential surface is represented as a sum of three potential interactions,

$$V_g(R_{C-I}, r, \theta) = V_a(R_{C-I}) + V_b(r) + V_c(\theta), \tag{5}$$

where the $V_a(R_{C-I})$ interaction in the R_{C-I} coordinate is taken from the CH_3I two-dimensional ground state potential recently reported by Alekseyev et al., and computed through multireference spin-orbit configuration interaction *ab initio* calculations.[9] In this *ab initio* potential the C−I equilibrium distance is $R_{C-I} = 4.04\ a_0$, and the calculated values of $D_e(CH_3 - I)$ and $D_0(CH_3 - I)$ are 2.432 eV and 2.26 eV, respectively, this latter quantity deviating $\sim$ 0.15 eV from the experimental value $D_0(CH_3 - I) = 2.41 \pm 0.02$ eV.[10] Since the R_{C-I} coordinate does not coincide with the Jacobi coordinate R used in the calculations, the $V_a(R_{C-I})$ potential in eq 5 is transformed to the (R, r, θ) coordinates in order to obtain a ground potential surface $V_g(R, r, \theta)$.

The $V_b(r)$ and $V_c(\theta)$ interactions are represented by harmonic oscillator functions,

$$V_b(r) = \frac{1}{2}k_b(r - r_0)^2 \ , \ V_c(\theta) = \frac{1}{2}k_c\theta^2. \tag{6}$$

In the case of $V_b(r)$ the force constant is $k_b = m\omega_b^2$, with $\omega_b = 1254$ cm^{-1} and $r_0 = 0.6555\ a_0$. For the $V_c(\theta)$ potential $k_c = \mu_c\omega_c^2$, with $\omega_c = 883$ cm^{-1} and the bending reduced mass μ_c being given by[1]

$$\frac{1}{\mu_c} = \frac{1}{m_I r_{C-I}^2} + \frac{1}{m_{H_3} r_{C-H_3}^2} + \frac{1}{m_C}\left(\frac{1}{r_{C-I}^2} + \frac{1}{r_{C-H_3}^2} + \frac{2}{r_{C-I}r_{C-H_3}}\right), \tag{7}$$

which by replacing the different distances and masses by their proper values gives the quantity $\mu_c = 1767.784$ amu.

Regarding the excited electronic states $^3Q_0$, $^1Q_1$, and the nonadiabatic coupling between them, in the simulations we have used the *ab initio* surfaces calculated by Xie at al.,[3] which are an improved version of the previous nine-dimensional surfaces of Amatatsu et al.[8] The surfaces and nonadiabatic couplings of refs 3 and 8 have the same functional form, only differing in the value of some of the parameters used to fit the *ab initio* points. Since the surfaces and couplings between them have been described in detail in ref 8, in the following we shall use the notation of that work when we refer to them. Specifically, we have used the diabatic potential surfaces V_1 and V_3 of ref 8, which correspond to the $^1Q_1(A')$ and $^3Q_0(A')$ electronic states, respectively, nonadiabatically coupled by the V_{13} coupling. The three surfaces V_1, V_3, and V_{13} are nine-dimensional ones, and in order to reduce their dimensionality, six out of the nine coordinates have been fixed at their equilibrium values. To this purpose the three C−H distances are fixed to the value $r_1 = r_2 = r_3 = 2.05266$ a_0. In addition, the angle ϕ between the plane containing the I−C bond and the plane containing one of the C−H bonds is fixed to 0°, and the three β angles (one of which is redundant) between the two planes containing C−H bonds are fixed to $\beta_1 = \beta_2 = \beta_3 = 120°$ (see Fig. 1 of ref 8. The resulting three-dimensional surfaces are transformed to the (R, r, θ) Jacobi coordinates employed in the simulations.

For the $^3Q_1$ excited state, the two-dimensional surface calculated *ab initio* by Alekseyev et al. was used.[9] This surface has no θ dependence, and this dependence was added as described elsewhere [Aguirre].

The initial state of CH_3I is calculated variationally on the ground electronic potential surface $V_g(R, r, \theta)$ by solving the Schrödinger equation

$$[\hat{T} + V_g(R, r, \theta)]\psi(R, r, \theta) = E\psi(R, r, \theta), \quad (8)$$

where $\hat{T}$ is expressed as in eq 1. A rigorous variational solution of eq 8 involves diagonalization of a extremely large Hamiltonian matrix, which becomes impractical. Thus, in order to make the problem tractable an adiabatic approximation was aplied.

In a first step the Schrödinger equation

$$\left[-\frac{\hbar^2}{2m}\frac{\partial^2}{\partial r^2}+\left(\frac{1}{2\mu R^2}+\frac{1}{2I_{CH_3}}\right)\hat{j}^2+V_g(R,r,\theta)\right]\varphi_j(R;r,\theta)=$$
$$\varepsilon_j(R)\varphi_j(R;r,\theta), \qquad (9)$$

where j denotes the vibrational quantum number, is solved for fixed values of the R coordinate in a uniform grid of 401 equidistant points in the range $3.5\,a_0 \leq R \leq 16.0313\,a_0$ with a step $\Delta R = 0.0313\ a_0$. Solution of eq 9 for each fixed R distance is obtained by representing the Hamiltonian matrix in a basis set in the r and θ coordinates and diagonalizing. The Fourier grid Hamiltonian (FGH) method[11] was used to represent the radial coordinate r in a grid of 33 equidistant points in the range $-1.6\,a_0 \leq r \leq 1.703\,a_0$, while a basis of 24 Legendre polynomials was used to represent the angular coordinate θ.

After solving eq 9 the functions $\varphi_j(R;r,\theta)$ and $\varepsilon_j(R)$ are obtained. Now, using $\varepsilon(R)$ as a potential-energy function we solve the equation

$$\left[-\frac{\hbar^2}{2\mu}\frac{\partial^2}{\partial R^2}+\varepsilon_j(R)\right]\chi_i^j(R)=E_i^j\chi_i^j(R), \qquad (10)$$

using again the FGH method. Again, i is the corresponding vibrational quantum number. The initial state of CH_3I is the ground vibrational state of the system obtained as outlined above, which is represented as

$$\psi_{i,j}(R,r,\theta)=\chi_i^j(R)\varphi_j(R_e;r,\theta), \qquad (11)$$

where R_e is the R equilibrium distance in the ground electronic potential surface.

Wave packet simulations

In the simulations CH_3I is excited from the $\tilde{X}^1A_1$ state to the three excited electronic states. The electric-dipole moment functions for all the transitions have been taken from the *ab initio* calculations of Alekseyev et al.,[12] and fitted to analytical functions, as described elsewhere.[13,14]

For the sake of simplicity, in the following we shall denote the four electronic states $\tilde{X}^1A_1$, $^3Q_0$, $^1Q_1$, and $^3Q_1$ involved in the photodissociation process by $|0>$,

$|1>$, $|2>$, and $|3>$, respectively, and correspondingly, their associated potential surfaces by $\hat{V}_0$, $\hat{V}_1$, $\hat{V}_2$, and $\hat{V}_3$. The nonadiabatic coupling between the excited states $|1>$ and $|2>$ will be denoted by $\hat{V}_{12}$. The states $|0>$ and $|1>$, $|2>$, and $|3>$, are radiatively coupled by the transition dipole moment and the radiation electric field $E(t)$, through the coupling $\mu_{01}E(t)$, $\mu_{02}E(t)$, and $\mu_{03}E(t)$, where μ_{0i} is the corresponding transition moment function.

The wave packet can be expressed as

$$\Phi(R,r,\theta) = \psi_0(R,r,\theta)|0> + \psi_1(R,r,\theta)|1> + \psi_2(R,r,\theta)|2> + \psi_3(R,r,\theta)|3> . \tag{13}$$

The time evolution of the amplitudes ψ_0, ψ_1, ψ_2, and ψ_3 on the four electronic potential-energy surfaces is governed by the following time-dependent coupled equations,

$$\begin{aligned} i\hbar\frac{\partial\psi_0}{\partial t} &= \hat{H}_0\psi_0 - \mu_{01}E(t)\psi_1 - \mu_{02}E(t)\psi_2 - \mu_{03}E(t)\psi_3, \\ i\hbar\frac{\partial\psi_1}{\partial t} &= \hat{H}_1\psi_1 - \mu_{10}E(t)\psi_0 + \hat{V}_{12}\psi_2, \\ i\hbar\frac{\partial\psi_2}{\partial t} &= \hat{H}_2\psi_2 - \mu_{20}E(t)\psi_0 + \hat{V}_{21}\psi_1, \\ i\hbar\frac{\partial\psi_3}{\partial t} &= \hat{H}_3\psi_3 - -\mu_{30}E(t)\psi_0, \end{aligned} \tag{14}$$

where $\hat{H}_i = \hat{T}_i + \hat{V}_i$ [being $\hat{T}_i$ expressed as in eq 1], $\mu_{i0} = \mu_{0i}$, and $\hat{V}_{21} = \hat{V}_{12}$. Solution of eqs 14 is subject to the initial condition $\psi_0(R,r,\theta,t=0) = \psi_{i,j}(R,r,\theta)$, $\psi_1(R,r,\theta,t=0) = \psi_2(R,r,\theta,t=0) = \psi_3(R,r,\theta,t=0) = 0$. The $E(t)$ is assumed to be a delta function at $t = 0$, independent of time. This implies that at $t = 0$ the initial vibrational state $\psi_0(R,r,\theta,t=0) = \psi_{i,j}(R,r,\theta)$ multiplied by the corresponding μ_{i0} transition dipole moment is excited to each of the three excited electronic states, and then propagated in time. This creates on each excited potential surface a wave packet that contains a wide range of energies. In this case eqs 14 simplify to

$$\begin{aligned} i\hbar\frac{\partial\psi_0}{\partial t} &= \hat{H}_0\psi_0, \\ i\hbar\frac{\partial\psi_1}{\partial t} &= \hat{H}_1\psi_1 + \hat{V}_{12}\psi_2, \end{aligned} \tag{15}$$

$$i\hbar\frac{\partial\psi_2}{\partial t} = \hat{H}_2\psi_2 + \hat{V}_{21}\psi_1,$$

$$i\hbar\frac{\partial\psi_3}{\partial t} = \hat{H}_3\psi_3,$$

In order to solve eqs 15 the wave packet is represented in a basis set consisting of a two-dimensional rectangular grid for the radial coordinates and an angular basis including 24 Legendre polynomials for the θ coordinate. The rectangular grid consists of 450 equidistant points in the R coordinate in the range $3.5\,a_0 \leq R \leq 16.0\,a_0$, and 32 equidistant points in the r coordinate distributed in the range $-1.6\,a_0 \leq r \leq 1.6\,a_0$. The wave packet is propagated using the Chebychev polynomial expansion method[15] to express the evolution operator. Propagation was carried out for 200 fs with a time step $\Delta t = 0.4$ fs. The wave packet is absorbed at the edge of the grid in the R coordinate after each propagation time step by multiplying each ψ_i packet by the function $\exp[-\alpha(R - R_{abs})^2]$, with $\alpha = 0.9\ a_0^{-2}$ and $R_{abs} = 13.0\ a_0$.

Calculation of observable magnitudes

Dissociation of CH_3I occurs through two fragmentation channels, namely CH_3 + I* and CH_3 + I, where the CH_3 fragment is produced with a distribution of internal states. In our model CH_3 is treated as a C−X pseudodiatomic molecule, which implies that only two internal degrees of freedom are considered, namely the C−X stretch vibration (which approximates the CH_3 umbrella mode) and the C−X rotation. The eigenstates associated with these internal modes of the C−X fragment are represented by the product $\chi_\nu^{(j)}(r)P_j(\cos\theta)$, where $P_j(\cos\theta)$ is a normalized Legendre polynomial and $\chi_\nu^{(j)}(r)$ are the solutions of the equation

$$\left[-\frac{\hbar^2}{2m}\frac{\partial^2}{\partial r^2} + \frac{j(j+1)\hbar^2}{2I_{CH_3}} + V_{CH_3}(r)\right]\chi_\nu^{(j)}(r) = E_{\nu,j}\chi_\nu^{(j)}(r), \quad (16)$$

being $V_{CH_3}(r)$ the CH_3 (or C−X) interaction potential at very large separation from the I atom,[8] and $E_{\nu,j}$ the energies associated with the eigenstates.

The probability (or cross section) of photodissociation of CH_3I is computed along time by projecting out the wave packet onto the corresponding fragment states. This

cross section is obtained by means of the method of Balint-Kurti et al.[16,17]

$$\sigma_v^{i,\nu,j}(E) = C\nu_0 k_{\nu,j}^{(i)} \left| \int_0^\infty < \chi_\nu^{(j)}(r) P_j(\cos\theta) | \psi_{i,v}(R_c, r, \theta, t') > e^{iEt'/\hbar} dt' \right|^2 , \qquad (17)$$

where C is a constant factor, $\nu_0 = \omega_0/2\pi$ is the incident photon frequency, $i = 1, 2, 3$ denotes the electronic state, R_c is a suitably large distance of the dissociation coordinate R located before the absorption region ($R_c = 12.99\ a_0$), E is the total energy of the system reached with the photon excitation, $E = E_i + \hbar\omega_0$ (being E_i the energy of the CH_3I initial state), and $k_{\nu,j}^{(i)}$ is given by

$$k_{\nu,j}^{(i)} = [2m(E - \epsilon\delta_{i1} - E_{\nu,j})]^{1/2}, \qquad (18)$$

being ϵ the spin-orbit splitting between the two electronic states of I and δ_{i1} the Kronecker delta. Now,

$$A_v^{i,\nu,j}(E) = \sqrt{C\nu_0 k_{\nu,j}^{(i)}} \int_0^\infty < \chi_\nu^{(j)}(r) P_j(\cos\theta) | \psi_{i,v}(R_c, r, \theta, t') > e^{iEt'/\hbar} dt'. \qquad (19)$$

References

(1) Guo, H. A Wave-Packet Study on Nonadiabatic Transition Dynamics in Photodissociation: The Importance of Parent Bending Motion. *J. Chem. Phys.* **1992**, *96*, 2731-2739.

(2) Guo, H. Three-Dimensional Photodissociation Dynamics of Methyl Iodide. J. Chem. Phys. **1992**, *96*, 6629–6642.

(3) Xie, D.; Guo, H.; Amatatsu, Y.; Kosloff, R. Photodissociation Dynamics of Rotational State Selected Methyl Iodide. *J. Phys. Chem. A* **2000**, *104*, 1009-1019.

(4) Shapiro, M.; Bersohn, R. Vibrational Energy Distribution of the CH_3 Radical Photodissociated from CH_3I. *J. Chem. Phys.* **1980**, *73*, 3810-3817.

(5) Shapiro, M. Photophysics of Dissociating CH_3I: Resonance Raman and Vibronic Photofragmentation Maps. *J. Phys. Chem.* **1986**, *90*, 3644-3653.

(6) Guo, H.; Schatz, G. C. Time-Dependent Dynamics of Methyl Iodide Photodissociation in the First Continuum. *J. Chem. Phys.* **1990**, *93*, 393-402.

(7) Heller, E. J. Photofragmentation of Symmetric Triatomic Molecules: Time Dependent Picture *J. Chem. Phys.* **1978**, *68*, 3891-3896.

(8) Amatatsu, Y.; Yabushita, S.; Morokuma, K. Full Nine-Dimensional Ab Initio Potential Energy Surfaces and Trajectory Studies of A-Band Photodissociation Dynamics: $CH_3I^* \rightarrow CH_3 + I$, $CH_3 + I^*$, and $CD_3I^* \rightarrow CD_3 + I$, $CD_3 + I^*$. *J. Chem. Phys.* **1996**, *104*, 9783–9794.

(9) Alekseyev, A. B.; Liebermann, H.-P.; Buenker, R. J.; Yurchenko, S. N. An Ab Initio Study of the CH_3I Photodissociation. I. Potential Energy Surfaces. *J. Chem. Phys.* **2007**, *126*, 234102.

(10) Eppink, A. T. J. B.; Parker, D. H. Energy Partitioning Following Photodissociation of Methyl Iodide in the A Band: a Velocity Mapping Study. *J. Chem. Phys.* **1999**, *110*, 832–844.

(11) Marston, C. C.; Balint-Kurti, G. G. The Fourier Grid Hamiltonian Method for Bound State Eigenvalues and Eigenfunctions. *J. Chem. Phys.* **1989**, *91*, 3571-3576.

(12) Alekseyev, A. B.; Liebermann, H.-P.; Buenker, R. J.; Yurchenko, S. N. An Ab Initio Study of the CH_3I Photodissociation. II. Transition Moments and Vibrational State Control of the I* Quantum Yields. *J. Chem. Phys.* **2007**, *126*, 234103.

(13) de Nalda, R.; Durá, J.; García-Vela, A.; Izquierdo, J. G.; González-Vázquez, J.; Bañares, L. A Detailed Experimental and Theoretical Study of the Femtosecond A-Band Photodissociation of CH_3I. *J. Chem. Phys.* **2008**, *128*, 244309.

(14) Rubio-Lago, L.; García-Vela, A.; Arregui, A.; Amaral, G. A.; Bañares, L. The Photodissociation of CH_3I in the Red Edge of the A-Band: Comparison Between Slice Imaging Experiments and Multisurface Wave Packet Calculations. *J. Chem. Phys.* **2009**, *131*, 174309.

(15) Tal-Ezer, H.; Kosloff, R. An Accurate and Efficient Scheme for Propagating the Time Dependent Schroedinger Equation. *J. Chem. Phys.* **1984**, *81*, 3967–3971.

(16) Balint-Kurti, G. G.; Dixon, R. N.; Marston, C. C. Time-Dependent Quantum Dynamics of Molecular Photofragmentation Processes. *J. Chem. Soc. Faraday Trans.* **1990**, *86*, 1741-1749.

(17) Balint-Kurti, G. G.; Dixon, R. N.; Marston, C. C. Grid Methods for Solving the Schroedinger Equation and Time Dependent Quantum Dynamics of Molecular Photofragmentation and Reactive Scattering Processes. *Int. Rev. Phys. Chem.* **1992**, *11*, 317.
